# Whole-genome sequencing reveals genomic diversity and selection signatures in Xia’nan cattle

**DOI:** 10.1186/s12864-024-10463-3

**Published:** 2024-06-05

**Authors:** Xingya Song, Zhi Yao, Zijing Zhang, Shijie Lyu, Ningbo Chen, Xingshan Qi, Xian Liu, Weidong Ma, Wusheng Wang, Chuzhao Lei, Yu Jiang, Eryao Wang, Yongzhen Huang

**Affiliations:** 1 College of Animal Science and Technology, Northwest A&F University, No. 22 Xinong Road, Yangling Shaanxi, 712100 Shaanxi People’s Republic of China; 2 Institute of Animal Husbandry, Henan Academy of Agricultural Sciences, Zhengzhou, 450002 Henan People’s Republic of China; 3 Biyang County Xiananniu Technology Development Co., Ltd, Zhumadian, 463700 People’s Republic of China; 4 Henan Provincial Livestock Technology Promotion Station, Zhengzhou, 450008 Henan People’s Republic of China; 5 Shaanxi Agricultural and Animal Husbandry Seed Farm, Shaanxi Fufeng, 722203 People’s Republic of China

**Keywords:** Xia’nan cattle, Genetic diversity, Population structure, Genetic signatures

## Abstract

**Background:**

The crossbreeding of specialized beef cattle breeds with Chinese indigenous cattle is a common method of genetic improvement. Xia’nan cattle, a crossbreed of Charolais and Nanyang cattle, is China’s first specialized beef cattle breed with independent intellectual property rights. After more than two decades of selective breeding, Xia’nan cattle exhibit a robust physique, good environmental adaptability, good tolerance to coarse feed, and high meat production rates. This study analyzed the population genetic structure, genetic diversity, and genomic variations of Xia’nan cattle using whole-genome sequencing data from 30 Xia’nan cattle and 178 published cattle genomic data.

**Result:**

The ancestry estimating composition analysis showed that the ancestry proportions for Xia’nan cattle were mainly Charolais with a small amount of Nanyang cattle. Through the genetic diversity studies (nucleotide diversity and linkage disequilibrium decay), we found that the genomic diversity of Xia’nan cattle is higher than that of specialized beef cattle breeds in Europe but lower than that of Chinese native cattle. Then, we used four methods to detect genome candidate regions influencing the excellent traits of Xia’nan cattle. Among the detected results, 42 genes (θπ and CLR) and 131 genes (*F*_ST_ and XP-EHH) were detected by two different detection strategies. In addition, we found a region in BTA8 with strong selection signals. Finally, we conducted functional annotation on the detected genes and found that these genes may influence body development (*NR6A1*), meat quality traits (*MCCC1*), growth traits (*WSCD1*, *TMEM68*, *MFN1*, *NCKAP5*), and immunity (*IL11RA*, *CNTFR*, *CCL27*, *SLAMF1*, *SLAMF7*, *NAA35*, and *GOLM1*).

**Conclusion:**

We elucidated the genomic features and population structure of Xia’nan cattle and detected some selection signals in genomic regions potentially associated with crucial economic traits in Xia’nan cattle. This research provided a basis for further breeding improvements in Xia’nan cattle and served as a reference for genetic enhancements in other crossbreed cattle.

**Supplementary Information:**

The online version contains supplementary material available at 10.1186/s12864-024-10463-3.

## Introduction

Domestic cattle include *Bos taurus*, *Bos indicus*, and their crossbreeds [1]. Previous research has divided domestic cattle around the world into five core populations based on geographic location: European taurine, Eurasian taurine, East Asian taurine, Chinese indicine, and Indian indicine [2, 47]. Chinese native cattle, due to the development of agricultural civilization, have historically been bred for plowing, and it resulted in Chinese native cattle exhibiting better environmental adaptability and tolerance to coarse feed compared to specialized beef cattle breeds [3, 4]. However, these local breeds notably lag behind specialized beef cattle in terms of meat production performance. In the 1980s, the demand for draft cattle gradually declined with the rapid advancement of agricultural mechanization. Concurrently, the market demand for beef cattle increased fast, necessitating improving both meat quantity and quality. To tap into the production potential of Chinese native cattle, breeders often introduced foreign bloodlines through hybridization to develop new breeds suitable for China. The Charolais, originating from France, are renowned as a large-sized beef cattle breed known for their rapid growth and high meat yield. Nanyang cattle, one of China’s five major native cattle breeds, are mainly distributed in Nanyang City, Henan Province. Previous study has shown that it belonged to the crossbreed of *Bos taurus* and *Bos indicus*. It has the advantages of the tall physique, fine meat quality, and more intramuscular fat, but also has the disadvantages of the slow growth rate and low slaughter rate [5, 48, 49]. Then, breeders in Henan province introduced Charolais cattle to crossbreed with the Nanyang cattle. After over two decades of selective breeding, they cultivated China’s first beef cattle breed with independent intellectual property rights. Xia’nan cattle possess several advantages, such as early maturity, rapid growth, good meat quality, and low dystocia rates, enhancing both beef production efficiency and the profitability of cattle farming for farmers.

In order to deeply study the genetic origin of excellent traits during Xia’nan cattle breeding, we sequenced 30 Xia’nan cattle’s genome and detected single nucleotide polymorphisms (SNPs) based on the reference genome of *Bos taurus* (ARS-UCD1.2). SNPs from Xia’nan cattle were compared with sites from beef cattle and Chinese native cattle previously collected.

## Result

### Sequencing mapping and SNP detection

This study generated 6,574,344,629 clean reads from 30 Xia’nan cattle whole-genome sequencing data. The reads were mapped to the reference genome (ARS-UCD1.2) and the average coverage of these samples is 11.64×. Then, 20,546,726 biallelic SNPs discovered in these 30 Xia’nan cattle were annotated. Functional annotation of SNP sites showed that the majority of SNPs were located in intergenic regions (58.8%) or intronic regions (37.5%). Exonic SNPs comprised 0.7% of the total SNPs, including 54,180 non-synonymous SNPs and 74,085 synonymous SNPs (Table S3). The total number of detected SNPs in the breeds is shown in Table S3. The count of SNPs in Xia’nan cattle is significantly lower than in Chinese indicine, Indian indicine, and Chinese native cattle (Nanyang cattle, Qinchuan cattle, and Jiaxian Red cattle) yet higher than in European taurine and Eurasian taurine. This distribution pattern of SNPs is similar to Pi’nan cattle, which is also a hybrid breed of Nanyang cattle in a previous study [6].

### Population structure analysis and genetic diversity analysis

**Fig. 1:**
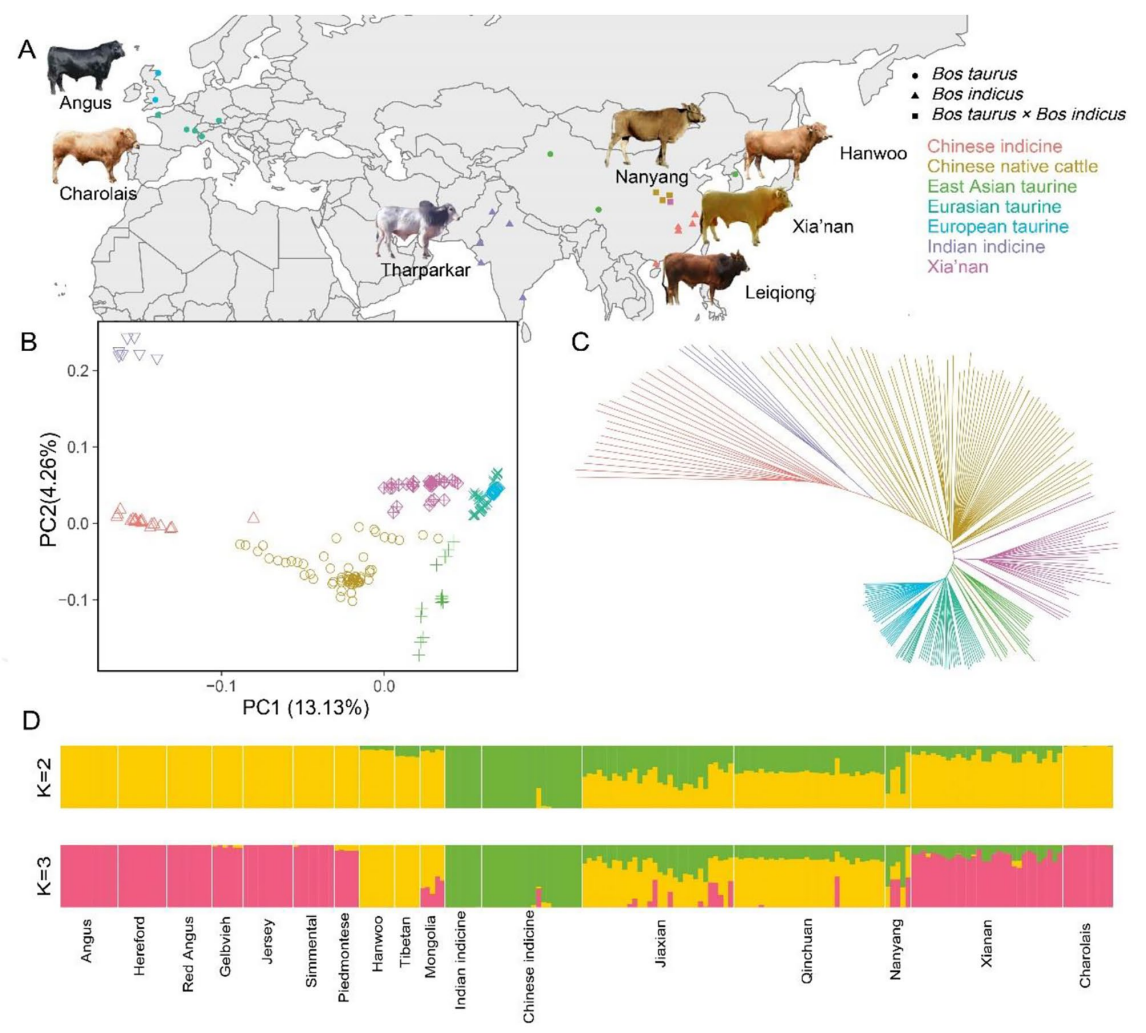
**A** Geographical distribution map of cattle breeds used in this study. **B** Principal component analysis of 17 cattle populations (208 individuals). **C** Neighbor-joining tree of the relationships in these populations. **D** Ancestry component analysis of these cattle breeds using ADMIXTURE with K = 2 and K = 3

In order to delve deeper into the genetic background of Xia’nan cattle, this study conducted ancestry estimating analysis and Principal Component Analysis (PCA) and built the Neighbor-Joining (NJ) tree. The results from NJ tree and PCA exhibit similar patterns, revealing distinct geographical clustering among cattle populations. The first Principal Component (PC) explained 13.13% of the whole genome variation and the second PC explained 4.26%. In the NJ tree, these cattle are clearly divided into different clusters according to five “core” populations [2]. Xia’nan cattle was positioned between Chinese native cattle and Eurasian taurine, closer to the Eurasian taurine (Fig. 1B and C). The result without Indian indicine is also shown in this study (Fig. S1), and it’s similar to Fig. 1B. In the ancestry estimating analysis, we showed the cases which the CV error (Table S4) is small, and the rest were shown in Fig. S2. When K = 2, There are clearly two components to the pedigree of these cattle: *Bos taurus* and *Bos indicus*. When K = 3, When K = 3, the Xia’nan cattle exhibited a higher resemblance to Charolais than Nanyang cattle, and it has more European taurine ancestry (Fig. 1D). because these cattle were divided into six reference groups and one target group, we also plotted the case at K = 4–7 (Fig. S1).

**Fig. 2:**
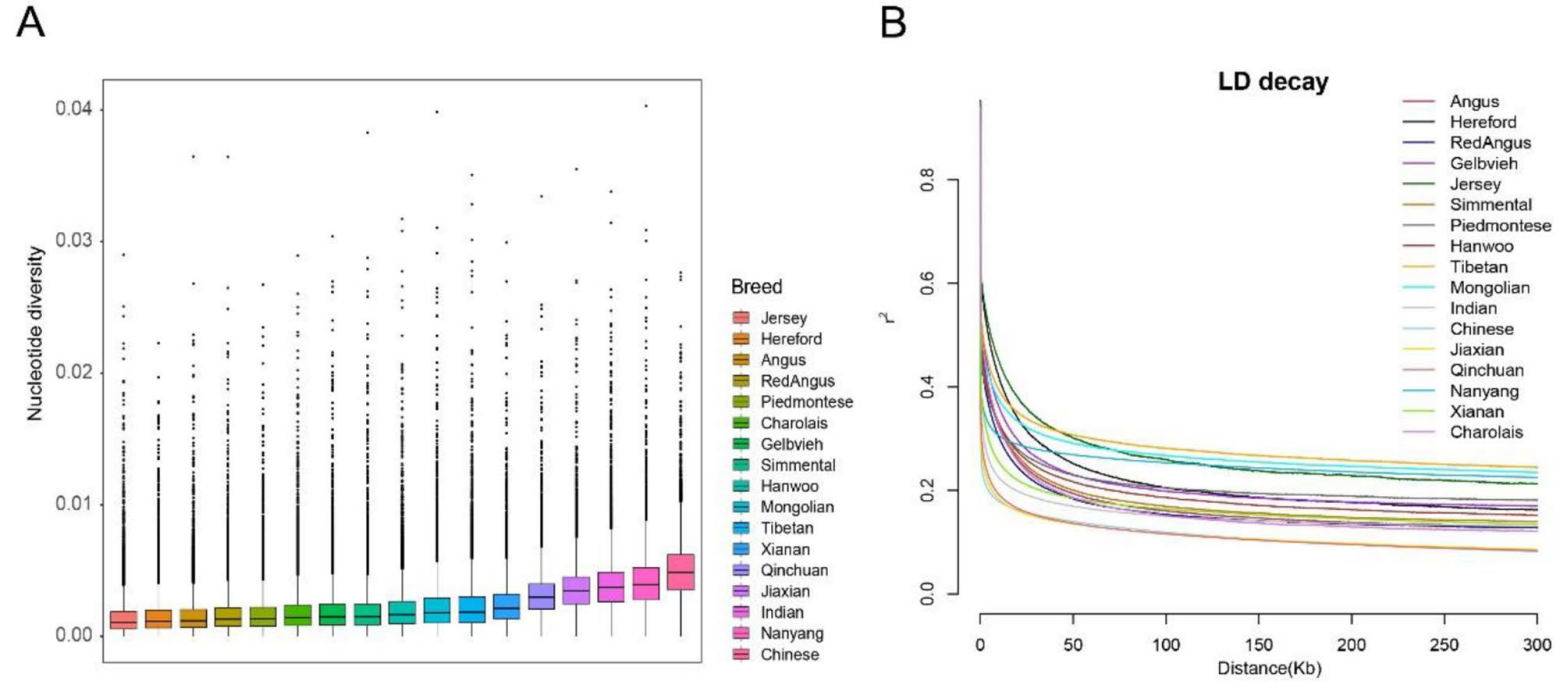
Genetic diversity analysis of these cattle. **A** Genome-wide distribution of nucleotide diversity of each breed in 50 kb windows with 50 kb steps. The horizontal line inside the box indicates the median of this distribution; box limits indicate the first and the thirds quartiles, points shows outliers. Data points outside the whiskers can be considered as outliers. **B** Genome-wide average LD decay estimated from each breed

To assess the genetic diversity of Xia’nan cattle, we calculated nucleotide diversity and linkage disequilibrium (LD) in Xia’nan cattle and other breeds. The results revealed that the highest nucleotide diversity was observed in Chinese indicine. Xia’nan cattle exhibited slightly higher nucleotide diversity compared to its paternal Charolais but lower than its maternal Nanyang cattle (Fig. 2A). Linkage disequilibrium analysis at the distance of 100 kb indicated that Jiaxian Red cattle and Qinchuan cattle had the lowest LD values, while Tibetan cattle exhibited the highest LD value, followed by Mongolian cattle. Xia’nan cattle showed higher LD levels than Charolais but lower than Nanyang cattle (Fig. 2B). In addition, we tested runs of homozygous (ROH) in these breeds and counted the distribution of ROHs of different lengths in different populations. We used the average of the number of ROHs in each breed to characterize the distribution pattern (Fig. S3). The results showed Chinese native cattle and Chinese indicine had more short ROHs (0.5-1 Mb and 1–2 Mb). Xia’nan cattle exhibited a pattern similar to that of the European taurine (Angus, Hereford and Red Angus).

### Genome-wide selective sweep test

**Fig. 3:**
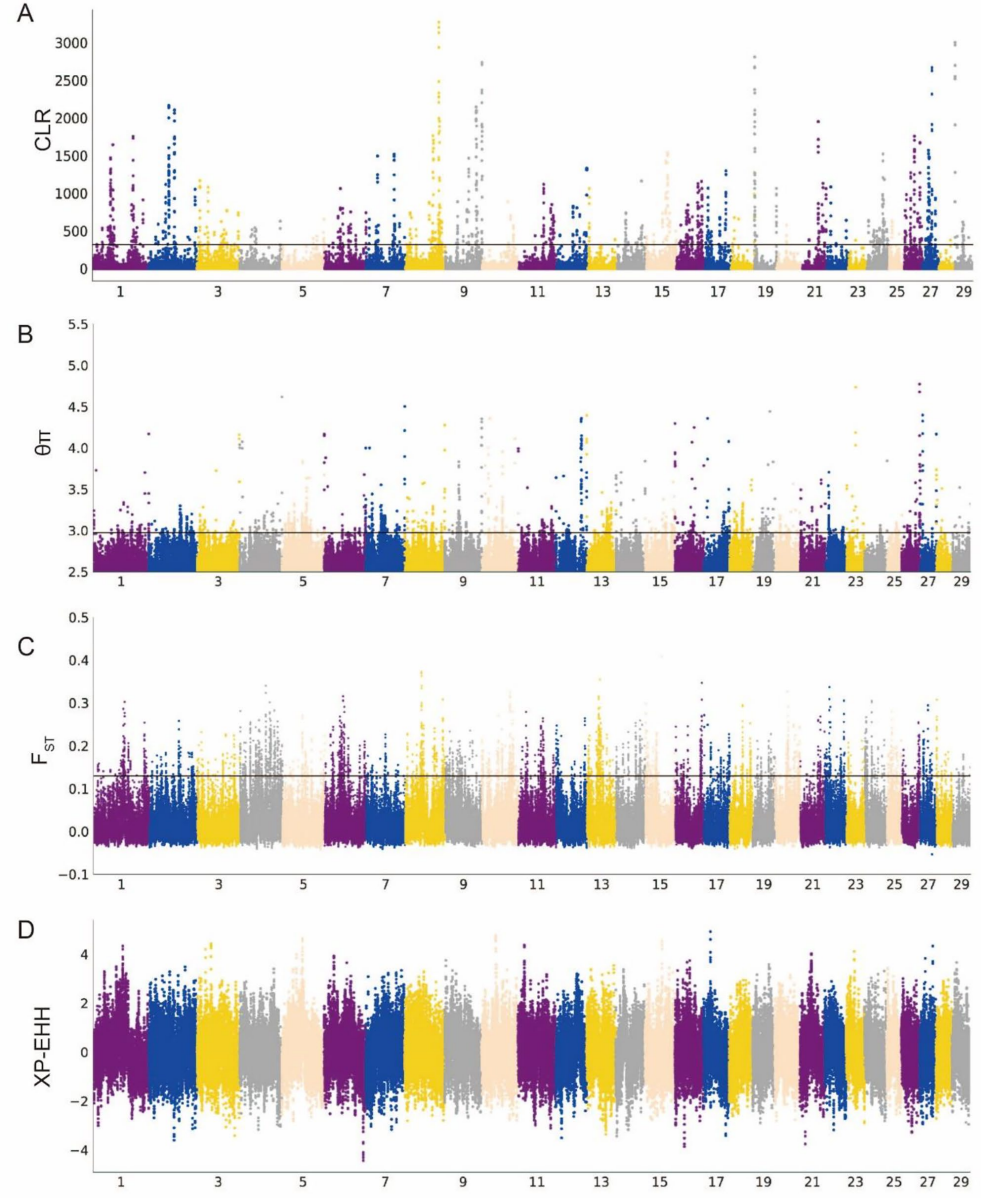
Manhattan plots of Analysis of the signatures of positive selection in the genome of Xia’nan cattle

We employed nucleotide diversity (θπ) analysis and composite likelihood ratio (CLR) methods to detect and select genomic regions associated with genetic diversity. Regions that showed high signals (top 1%) by both methods were considered candidate selection regions (Fig. 3A and B). In Xia’nan cattle, a total of 1184 genes (θπ) and 484 genes (CLR) exhibiting selection features were identified, with 40 genes overlapping (Tables S5 and S6). Functional enrichment analysis using Kyoto Encyclopedia of Genes and Genomes (KEGG) pathways and Gene Ontology (GO) was conducted for these overlapping genes. The top term enriched by KEGG is “Cytokine-cytokine receptor interaction” (*P*-value = 0.006), Involving 3 genes (*IL11RA*, *CNTFR*, *CCL27*). The GO terms with a significant (*P*-value < 0.05) association in these overlapped genes include cytokine binding (GO:0019955), regulation of transcription, DNA-templated (GO:0006355), receptor complex (GO:0043235), and cytokine receptor activity (GO:0004896). Most terms are closely associated with the immune (Table S7 and S8).

Subsequently, we conducted fixation index (*F*_ST_) and cross-population extended homozygosity (XP-EHH) tests between the Xia’nan cattle and Nanyang cattle, and the top 1% of all sites are positive sites (Fig. 3C and D). The *F*_ST_ method detected 1344 genes, while the XP-EHH test identified 1355 genes (Tables S9 and S10). Among these, 131 genes were detected by both methods and confirmed as potential candidate genes specific to Xia’nan cattle. The KEGG and GO analysis results of these genes are shown in Table S12. Additionally, some regions with strong signals of selection contained known genes associated with traits of interest, such as growth (*WSCD1*, *TMEM68*, *MFN1*, *NCKAP5*), environmental adaptation (*ITPR2*, *GBA3*), fat metabolism (*CRTC1*, *HSPA12A*), and meat quality (*MCCC1*).

**Fig. 4:**
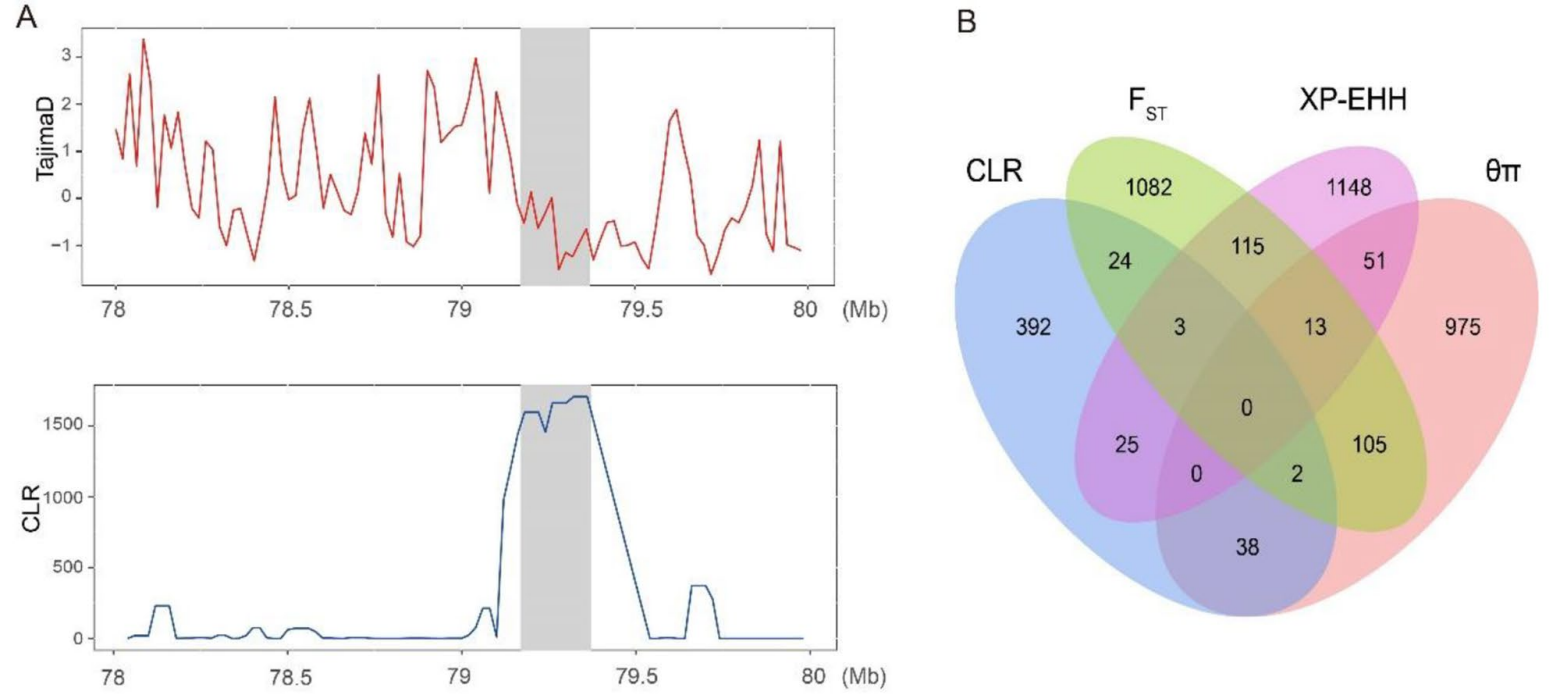
**A** Tajima’s D and CLR values on BTA8: 79.1–79.4 Mb region. **B** Venn diagram showing the overlapping gene counts among θπ, CLR, *F*_ST_ and XP-EHH.

In addition, we observed a significant peak on BTA8: 79.1–79.4 Mb, which was found by three methods (CLR, θπ, and *F*_ST_) (Fig. 4A and B). This region includes two immune-related genes: *NAA35* and *GOLM1*.

## Discussion

Population genetics and genetic diversity analyses can aid in assessing genetic resources and breed improvement in Xia’nan cattle. The results of ADMIXTURE software analysis show that the ancestry composition of Xia’nan cattle is more similar to that of Charolais cattle, with most European taurine ancestry. This tiny genetic contribution from Nanyang cattle results higher nucleotide diversity of the Xia’nan cattle genome than European taurine’s. However, the genomic diversity of Xia’nan cattle is lower than that of Chinese native cattle. It may be due to the founder effect or the recent intensive artificial selection in this population of Xia’nan cattle. The LD pattern of each variety was basically consistent with the results of nucleotide diversity. The higher LD level in Xia’nan cattle than in Charolais indicated that there may be more breeding in Xia’nan cattle. The differing LD imbalance intensity in Nanyang cattle compared to other Chinese local breeds might stem from a smaller sample size. The collected samples have undergone stronger artificial selection, emphasizing the rich variation sites and genetic resources inherent in Chinese native cattle breeds. The distribution pattern of ROHs can illustrate the breeding history of the cattle. The ROH distribution pattern of Xia’nan cattle is similar to that of European taurine, reflecting the recent intensive artificial selection in this breed. The pattern of Nanyang cattle and other Chinese native cattle breeds showed large differences, probably because the sample count of Nanyang cattle was too less.

Chinese native cattle are often better resistant and more adaptable than foreign beef breeds. In our selective sweep analysis of Xia’nan cattle, we found several genes associated with immune function, including *IL11RA*, *CNTFR*, and *CCL27*, within the “cytokine-cytokine receptor interaction” pathway. Interleukin-11 receptor alpha (IL11RA) is the receptor of IL-11. In mice, the specific expression of *IL-11* transgenes in fibroblasts or the injection of IL-11 leads to heart and kidney fibrosis, ultimately resulting in organ failure. Conversely, the deletion of IL11RA1 offered protection against these diseases [7]. Recent research has shown that inhibition of *CNTFR* could diminish the inhibitory effects of CLCF1 on mitochondrial biogenesis and thermogenesis [8]. The CC chemokine receptor 27 (CCL27), predominantly expressed by skin keratinocytes, plays a crucial role in the establishment of resident lymphocytes and maintaining immune balance in barrier tissues. It’s closely linked to normal skin and hair follicle development [9].

The candidate genes *SLAMF1* and *SLAMF7* are also related to immunity in cattle in previous research [10]. SLAMF1 and SLAMF7 both belong to the signaling lymphocytic activation molecule (SLAM) family, typically regarded as potential targets for inflammation and autoimmune diseases [11, 12]. Previous studies have identified the SLAMF1 as a crucial negative regulator in humoral immune responses [13], initiating signal transduction networks across various immune cells [14]. Meanwhile, the SLAMF7 plays a significant role in macrophage hyperactivation and may have critical implications in modulating T-cell functionality within the tumor microenvironment [15]. Additionally, SLAMF7 is involved in regulating B-cell responses and adaptive immunity [16], potentially modulating susceptibility to autoimmune conditions in the central nervous system [17]. The strong selection region (BTA8: 79.1–79.4 Mb) that we found by three methods contains two genes: N-Alpha-Acetyltransferase 35 (*NAA35*) and Golgi membrane protein 1 (*GOLM1*). A previous study has shown that the cytokine quantitative trait locus (QTL), including the *NAA35 - GOLM1*, significantly regulates the production of interleukin-6 in response to various pathogens [18].

These findings may be linked to the high disease resistance observed in Nanyang cattle and potentially contribute to Xia’nan cattle’s superior disease resistance compared to Charolais cattle [19]. Additionally, among the positively selected genes, we discovered *NR6A1*, a gene associated with mammalian trunk development and vertebral count [20, 21]. It may serve as a candidate locus influencing the body size of Xia’nan cattle.

We discovered lots of genes when comparing selection signals between Xia’nan and Charolais cattle. By searching the literature, we found that some of these genes influence important traits, for example, growth traits (*WSCD1*, *TEME68*, *MFN1*, *NCKAP5*), fat deposition (*CRTC1*, *HSPA12A*), environmental adaptation (*ITPR2*, *GBA3*), and meat quality (*MCCC1*). The WSC Domain Containing 1 (*WSCD1*) gene encodes a protein exhibiting sulfotransferase activity and playing a role in glucose metabolism. This gene has been identified to be associated with daily feeding time and feeding rate in the White Duroc × Erhualian F population in a GWAS study [22], and it was linked to three reproductive periods’ body size in Simmental beef cattle in another GWAS study [8]. Transmembrane protein 68 (*TEME68*) was found that it may be associated with feed intake and growth phenotypes in cattle [23]. Mitochondrial fusion protein 1 (MFN1) is a key regulator of mitochondrial fusion in mammalian cells, playing a pivotal role in maintaining the stability of mitochondrial morphology. The copy number of this gene was found to be related to the growth traits of beef cattle [24]. NCK-associated protein 5 (*NCKAP5*) was found to be potentially associated with important phenotypes in Limousin cattle [25]. CREB-regulated transcription coactivator 1 (*CRTC1*) and Heat shock protein 12 A (*HSPA12A*) can respectively regulate adipocyte differentiation and fat metabolism through the PPARγ pathway [26, 27]. Inositol 1,4,5-Trisphosphate receptor type 2 (*ITPR2*) and Glucosylceramidase Beta 3 (*GBA3*) were reported as high-altitude adaptation genes [28–30]. Methylcrotonyl-CoA carboxylase 1 (*MCCC1*) was identified as a candidate gene for pork meat quality through weighted gene co-expression network analysis [31].

These genes, which have been found to be related to immunity, fat deposition, meat quality, and adaptability, may be the components of the genetic basis of Nanyang cattle for good adaptability, excellent disease resistance, and tender meat quality. These screened genes may all play important roles in the formation of Xia’nan cattle’s excellent traits and can be used as candidate genes for breeding of beef cattle.

## Conclusions

This study offers a thorough insight into genomic variations of Xia’nan cattle through Whole Genome Sequencing (WGS) data analysis. The exploration of population structure and genetic diversity in Xia’nan cattle will provide valuable guidance for developing informed and effective breeding strategies. Furthermore, we identified some candidate genes that may play crucial roles in growth traits, immune responses, and meat quality in beef cattle. Xia’nan cattle stand as a successful example of improvement among Chinese native cattle breeds, and unraveling the genetic factors behind their superior traits is pivotal for further enhancements within this breed and the development of novel breeds.

## Methods

### Sample and sequencing

We collected 30 blood samples of Xia’nan cattle from the Xia’nan breeding farm in Henan province (Table S1). The cattle were not treated harshly during sampling and were released after sampling. We employed the standard phenol-chloroform method for blood DNA extraction [32].

After assessing the DNA quality, gene libraries were assembled, each with an average fragment size of 300 bp per sample. The sequencing was carried out by BGI using the DNBSEQ-T7 sequencer.

To better elucidate the genetic variations in Xia’nan cattle, we expanded our sample collection by acquiring an additional 178 samples from five “core” cattle populations [2]. These samples encompass European taurine (Angus (*n* = 11), Hereford (*n* = 10), Red Angus (*n* = 9)), Eurasian taurine (Chariolais (*n* = 10), Gelbvieh (*n* = 6), Jersey (*n* = 10), Piedmontese (*n* = 5), and Simmental (*n* = 8)), East Asian taurine (Hanwoo (*n* = 7), Mongolia (*n* = 5), Tibetan (*n* = 5)), Chinese indicine (Guangfeng cattle (*n* = 4), Leiqiong cattle (*n* = 3), Ji’an cattle (*n* = 4), Jinjiang cattle (*n* = 4), Wannan cattle (*n* = 5)), Indian indicine (Gir (*n* = 2), Haryana (*n* = 1), Nelore (*n* = 1), Sahiwal (*n* = 1), Tharparkar (*n* = 1), unidentified breed (*n* = 1)) and Chinese native cattle ((Nanyang (*n* = 5), Jiaxian (*n* = 30), Qinchuan (*n* = 30)) (Table S2).

### Reads mapping, SNP calling, and annotation

The Trimmomatic software (version 0.38) [33]was utilized for trimming sequence reads, employing the following parameters: “LEADING:20, TRAILING:20, SLIDINGWINDWOE: 3:15, AVGQUAL:20, MINLEN:35, PHRED33”. The clean reads were aligned to the reference genome ARS-UCD1.2 by “bwa mem” software (version 0.7.13-r1126) with default parameters. Picard software (http://broadinstitute.github.io/picard) was used to remove PCR duplicates. After these, we used the “HaplotypeCaller”, “GenotypeGVCFs”, and “SelectVariants” modules in the Genome Analysis Toolkit (GATK, version 4.1.8.1) to detect SNPs and used the “VariantFiltration” module to filter SNPs with the parameters (QD < 2.0, QUAL < 30.0, SOR > 3.0, FS > 60.0, MQ < 40.0, MQRankSum < -12.5, and ReadPosRankSum < -8.0) [34]. Finally, BCFtools [35] was used to retain SNP sites with a genotype missing rate less than 0.1 and a minimum minor allele count greater than 2 (F_MISSING < 0.1 & MAC > 2). We employed ANNOVAR [30] software to annotate the functions of each SNP based on the *Bos taurus* reference genome annotation file (https://ftp.ncbi.nlm.nih.gov/genomes/all/GCF/002/263/795/GCF_002263795.1_ARS-UCD1.2/GCF_002263795.1_ARS-UCD1.2_genomic.gff).

### Population genetic analysis

We used PLINK (version 1.90) [36] to remove the SNPs with high LD. The parameter is “--indep-pair-wise 50 5 0.2”. PopLDdecay [37] software was used to calculate and visualize Linkage disequilibrium (LD) decay with physical distance between SNPs. PCA was conducted using the “SmartPCA” module within Eigensoft (v6.1.4) [38]. For ancestry estimation analysis, ADMIXTURE (version 1.3) [39] was employed with the kinship (K-value) ranging from 2 to 7, and TBtools [40] was used for visualization. We utilized the matrix of pairwise genetic distances supplied by PLINK was used to construct an unrooted evolutionary tree. The visualization of the evolutionary tree was carried out using MEGA11 [41] and FigTree v1.4.4 (http://tree.bio.ed.ac.uk/software/figtree/). We detected ROHs by PLINK with the following parameters: (1) the size of the window of 50 SNPs (--homozyg-window-snp 50); (2) required minimum density (--homozyg-density 50); (3) number of heterozygotes allowed in a window (--homozyg-window-het 3); (4) the number of missing calls allowed in window (--homozyg-window-missing 5). ROHs were divided into four categories based on length: 0.5-1 Mb, 1–2 Mb, 2–4 Mb, > 4 Mb.

### Selective sweep identification

In order to reveal the signatures of selection influenced by artificial selection and genetic adaptation to the local environment, we employed various strategies to detect the genome selection signals of Xia’nan cattle. Within the Xia’nan cattle population, the composite likelihood ratio (CLR) method [42] and the nucleotide diversity (θπ) were employed. The value for nucleotide diversity in the plot is -log_10_(θπ). We used VCFtools [43] software to estimate the whole-genome nucleotide diversity using the sliding window approach utilizing window sizes of 50 kb and steps of 20 kb. SweepFinder2 [44] was employed to calculate the CLR for every 50 kb window. Empirical *P*-values of these windows were calculated. The regions with the empirical *P*-value in the top 1% are positive regions, and the genes detected by both methods are regarded as candidate genes.

Furthermore, fixation index (*F*_ST_) and cross-population extended haplotype homozygosity (XP-EHH) analyses were conducted to compare Xia’nan cattle with Nanyang cattle. *F*_ST_ analysis was carried out by VCFtools with the same window size as CLR analysis. In the XP-EHH selection scans, the statistics were derived from the average of standardized XP-EHH scores calculated for every 50 kb region by SELSCAN V1.1 [45] software. The directional XP-EHH score indicated selection: a positive score suggested potential selection in Xia’nan cattle, while a negative score implied potential selection in the reference population. A significance threshold of *P*-value < 0.01 was applied to identify noteworthy genomic regions. Genes identified through both methods were considered as candidates for positive selection.

To further understand the functions and signaling pathways associated with these candidate genes, we used KOBAS (version 3.0) [46] to get enrichment pathways by GO and KEGG.

### Electronic supplementary material

Below is the link to the electronic supplementary material.

Supplementary Material 1

Supplementary Material 2

Supplementary Material 3

Supplementary Material 4

## Data Availability

Sequences are available from the Sequence Read Archive (SRA) database. Bioproject accession number is PRJNA1058368.
