# Supplementary figures and images for: Whole-genome sequencing reveals genomic diversity and selection signatures in Xia’nan cattle
Source: BMC Genomics. 2024 Jun 5;25:559. doi: 10.1186/s12864-024-10463-3 (PMC11151506; doi:10.1186/s12864-024-10463-3)

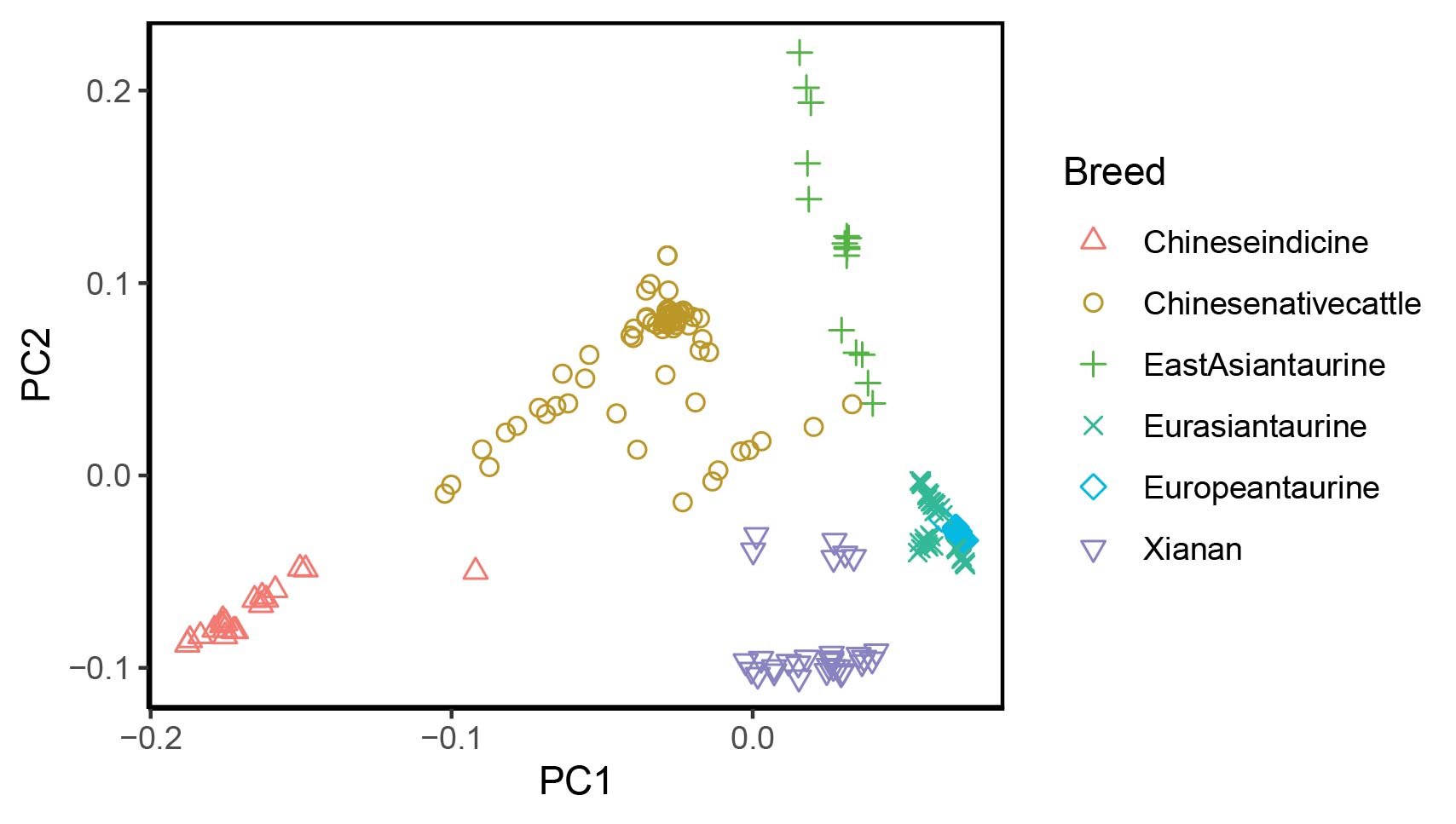

Supplement: Supplementary file 2 — Supplementary Material 2 [file 12864_2024_10463_MOESM2_ESM.jpg]

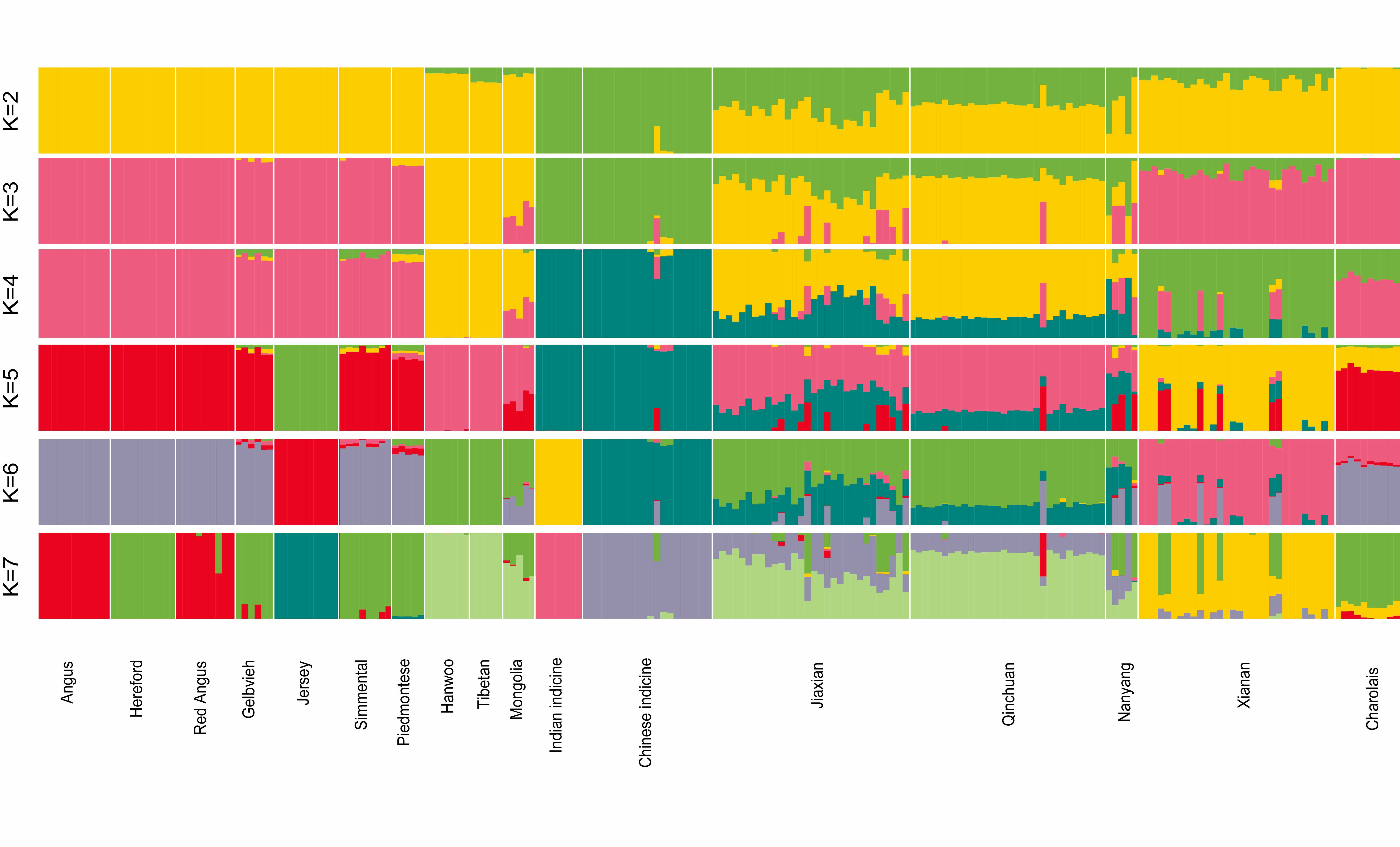

Supplement: Supplementary file 3 — Supplementary Material 3 [file 12864_2024_10463_MOESM3_ESM.jpg]

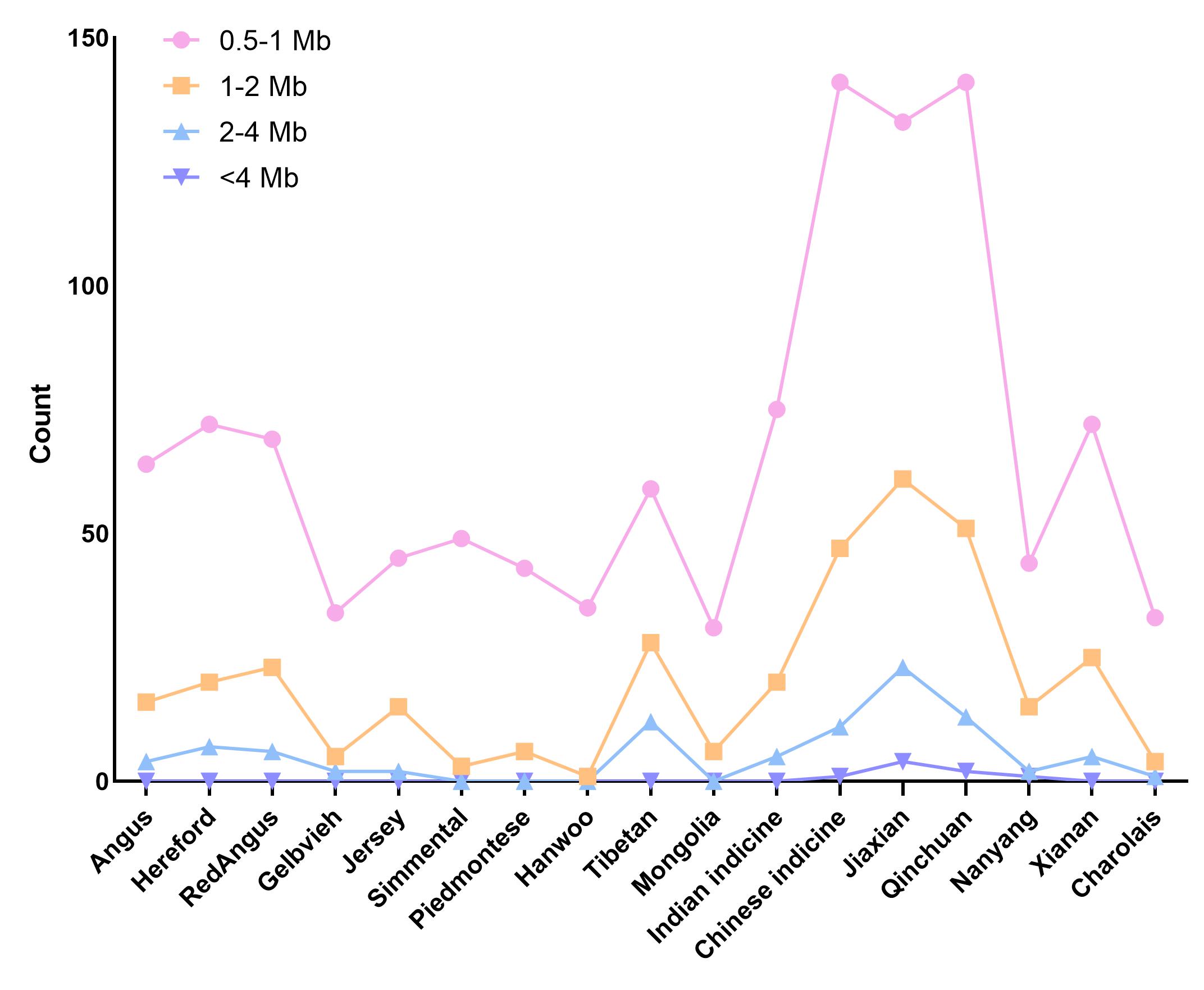

Supplement: Supplementary file 4 — Supplementary Material 4 [file 12864_2024_10463_MOESM4_ESM.jpg]
